# Voxel-Wise Quantitative Mapping of the Brain Association Ability

**DOI:** 10.3389/fnins.2021.746894

**Published:** 2021-10-15

**Authors:** Kai Chen, Lijie Wang, Jianguang Zeng, Ai Chen, Zhao Gao, Jiaojian Wang

**Affiliations:** ^1^School of Automation Engineering, University of Electronic Science and Technology of China, Chengdu, China; ^2^School of Computer Science and Engineering, University of Electronic Science and Technology of China, Chengdu, China; ^3^School of Economics and Business Administration, Chongqing University, Chongqing, China; ^4^Department of Pediatric Neurology-Gastroenterology and Newborn Screening Center, Sichuan Provincial Maternity and Child Health Care Hospital, Affiliated Women’s and Children’s Hospital of Chengdu Medical College, Chengdu, China; ^5^Key Laboratory for NeuroInformation of the Ministry of Education, School of Foreign Languages, University of Electronic Science and Technology of China, Chengdu, China; ^6^State Key Laboratory of Primate Biomedical Research, Institute of Primate Translational Medicine, Kunming University of Science and Technology, Kunming, China

**Keywords:** association index, association cortex, voxel-level, resting-state, functional connectivity

## Abstract

The association cortices of the brain are essential for integrating multimodal information that subserves complex and high-order cognitive functions. To delineate the changing pattern of associative cortices can provide critical insight into brain development, aging, plasticity, and disease-triggered functional abnormalities. However, how to quantitatively characterize the association capability of the brain is elusive. Here, we developed a new method of association index (Asso) at the voxel level to quantitatively characterize the brain association ability. Using the Asso method, we found high Asso values in association cortical networks, and low values in visual and limbic networks, suggesting a pattern of significant gradient distribution in neural functions. The spatial distribution patterns of Asso show high similarities across different thresholds suggesting that Asso mapping is a threshold-free method. In addition, compared with functional connectivity strength, i.e., degree centrality method, Asso mapping showed different patterns for association cortices and primary cortices. Finally, the Asso method was applied to investigate aging effects and identified similar findings with previous studies. All these results indicated that Asso can characterize the brain association patterns effectively and open a new avenue to reveal a neural basis for development, aging, and brain disorders.

## Introduction

The association cortex that plays a pivotal role in integrating multimodal information for high-order cognitive functions matures later during development while ages earlier during aging than primary cortices (Tomasi and Volkow, 2012; Gilmore et al., 2018). Degeneration of association cortices makes the brain more vulnerable to neuropsychiatric disorders such as schizophrenia, Alzheimer’s disease, and so on (Douaud et al., 2014). Moreover, during evolution and development, the association cortices expand much more greatly than other areas (Hill et al., 2010). Thus, delineating the association ability of the brain is fundamental to understand the mechanism of the brain evolution, development, aging, and the disease state.

Many brain structures have been documented as relay stations responsible for information exchange by receiving input signals and sending out instructions to other cortical or subcortical areas. For example, the thalamus that is located in the middle of the brain plays a relay role in the information transmission between the brain and the rest of the nervous system in the body (Steriade and Llinas, 1988; Haber and McFarland, 2001; Hwang et al., 2017; Redinbaugh et al., 2020). The posterior parietal cortex transmits the visual information received from the occipital cortex into the motor or prefrontal cortex to facilitate visually guided motor or attention or language processing (Eskandar and Assad, 1999; Corbetta et al., 2000; Iacoboni, 2006; Schluppeck et al., 2006; Wang et al., 2012, 2015, 2016b, 2017; Konen et al., 2013; Wang J. et al., 2019). However, how to quantify the brain association ability is still lacking.

Several data-driven approaches were developed to map the hub regions to reflect the association ability of the brain. To define the brain hubs, Buckner et al. (2009) introduced functional connectivity strength (FCS), i.e., degree centrality or degree in graph theory. Tomasi and Volkow (2010) proposed a functional connectivity density (FCD) mapping method to identify functional hubs by counting the number of connections with neighboring voxels beyond a predefined threshold in the human brain using a region-growing approach. Although both FCS and FCD can map the brain hubs, the two measures only count the connected edges of the target voxel with other voxels but neglect whether or not its connected neighborhoods are also directly interconnected to each other. Of note, the interconnective state of neighboring voxels will degrade the importance of the hub regions in information transition. More importantly, not all the association areas in the brain are hubs. Thus, it is imperative to develop a new approach to quantify the association ability of each voxel or each brain area.

In this study, to quantitatively characterize the association ability, we developed a new method using association index (Asso) to measure the association ability of each voxel of the brain directly. Moreover, we examine the robustness and effectiveness of Asso by testing different threshold values, comparing with FCS, and to explore the aging effects.

## Materials and Methods

### Resting-State Functional MRI Data and Preprocessing

A large cohort of public adult lifespan MRI data (Southwest University Adult Lifespan Dataset, SALD) was accessed through the f1000 project^1^. Out of 494 subjects, 262 healthy subjects (101 males/161 females between 19 and 75 years old; mean and standard deviation of age = 42.03 ± 16.73 years) with high-quality resting-state functional MRI (rs-fMRI) were finally used in this study. Exclusion criteria include excessive head motion (>2 mm or 2° in any direction or frame displacement >0.5) and insufficient images (the sum of deleted volumes per subject >116). The subjects were instructed to close their eyes and stay awake and not think of anything while scanned in an MRI scanner of Siemens 3T Tim Trio with echo planar imaging sequence (TR = 2,000 ms, TE = 30 ms, voxel size = 3.4 × 3.4 × 3 mm^3^ with 1-mm gap, 32 axial slices, and 242 volumes). All subjects were given informed written consents approved by the Institutional Review Board. See more details about subjects and rs-fMRI scanning parameters in Wei’s study (Wei et al., 2018).

The rs-fMRI data were pre-processed as follows: (1) The first 10 volumes were discarded to facilitate magnetization equilibrium. (2) All the remaining volumes were realigned to the first volume to correct head motion. (3) Normalize to the standard EPI template in MNI space. (4) Friston 24-parameter model of head motion, white matter, cerebrospinal fluid, and global mean signals were regressed out. (5) A temporal band-pass of 0.01–0.1 Hz was used in filtering. Scrubbing was further used to eliminate the bad images (before two time points and after one time points) exceeding the preset criteria [frame displacement (FD) < 0.5] for excessive motion.

### Association Index Mapping

**FIGURE 1:**
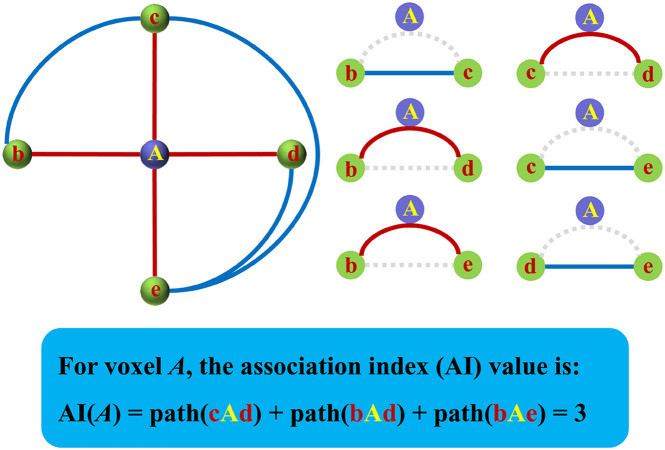
The diagram for the calculation of the association index (Asso). The Asso was defined as the total number of functional connections of this voxel to all other voxels of the rest brain above a predefined threshold minus the number of interconnected functional connections of the rest brain above the same threshold.

The Asso for a specific voxel was defined as the result of the total number of functional connections of this voxel to all other voxels minus that of interconnected functional connections of all the other voxels. The strength of all connections counted in should stay above the same threshold *r*. The specific steps of mapping are as follows: First, the whole-brain functional connectivities defined with Pearson’s correlation coefficients were calculated for a specific voxel. Then, a predefined correlation coefficient (e.g., *r* = 0.2, or 0.25, or 0.3, etc.) was used to identify the number (*N*) of functional connectivities with higher coefficients than this threshold, and the weak connections yielded by signal noises can be excluded under this threshold (Buckner et al., 2009; Wu H. et al., 2016; Liu et al., 2018; Song et al., 2020). After identifying the eligible connections and corresponding voxels, the number (*K*) of these functional connectivities among the identified voxels was further calculated. Finally, the Asso for this specific voxel was defined as the following formula (1). The same procedures were performed for all the voxels of the whole brain to obtain a whole-brain Asso map for each subject. Figure 1 shows the diagram for the calculation of Asso. The Asso maps were normalized using Z-transformation and were smoothed with a Gaussian kernel of 6-mm full width at half maximum for statistical analysis. A one-sample *t*-test was used to identify the whole brain distribution of Asso with the Z-transformed maps across all the subjects.


(1) $$A\,s\,s\,o=\frac{N\times \left(N-1\right)-2\times K}{2}$$

### Association Index Distribution in Cortical Networks

The brain cortex was subdivided into seven cortical networks, including the ventral attention network (vANT), dorsal attention network (vANT), default mode network (DMN), frontoparietal network (FPN), somatomotor network (Mot), visual network (Vis), and limbic network (LMB). The mean Asso values within each of these networks were calculated to determine the Asso distribution pattern of functional neural networks.

### Threshold Effects on Association Index

A predefined threshold was used to determine the real functional connectivities and exclude weak connections resulting from signal noises. We mapped the Asso referring to threshold *r* values of 0.2, 0.25, and 0.3, respectively, to explore the effects of different threshold values and calculated spatial correction analyses using Pearson’s correlation coefficients to reveal the similarity or dissimilarity.

### Comparisons Between Association Index and Functional Connectivity Strength

Although the FCS method employed a similar approach to map the brain hubs, it overlooks the directly interconnected neighbors. To show the difference between the Asso method and FCS, we calculated the whole-brain maps via both Asso and FCS for all the subjects at the same predefined threshold (*r* = 0.2) and normalized to z-scores. A paired *t*-test was used to compare Asso and FCS with the significant level of *p* < 0.05 and voxels >50 corrected by false discovery rate (FDR).

### Aging Effects Revealed by Association Index

Finally, we used the Asso method to further test whether Asso is an effective tool to reveal the aging effects on the association ability of the brain. To validate the specificity, we also included the FCS method for comparisons. The Asso and FCS maps were computed at the threshold of 0.2. The voxel-wise correlation analyses between ages and, respectively, Asso and FCS were performed. The significant correlations were determined using FDR correction with *p* < 0.05 and a minimum of 50 voxels.

## Results

### Association Index Distribution in Cortical Networks

**FIGURE 2:**
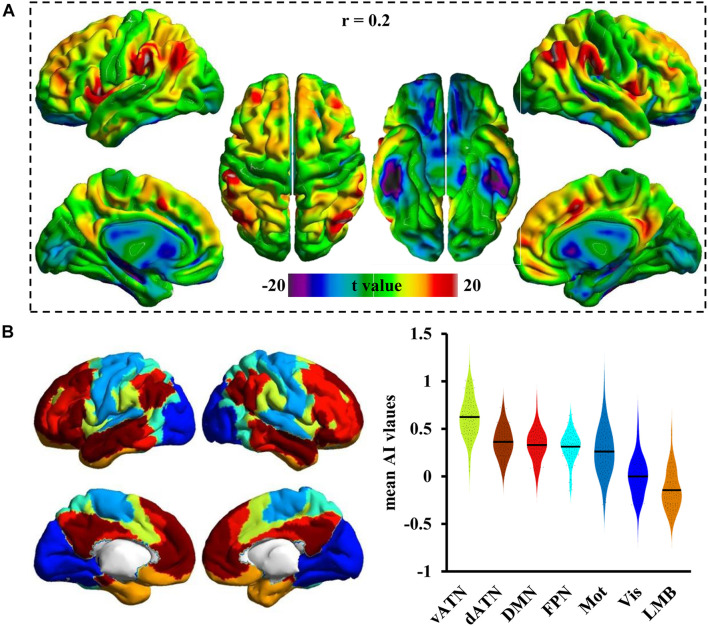
The distribution pattern of the Asso in the cortex. **(A)** The whole brain Asso map was calculated at a predefined threshold of 0.2, and one-sample *t*-test was used to identify the distribution pattern in cortex. **(B)** The human cortex was divided into seven subnetworks, and the mean Asso value was calculated for each network. The Asso distributions from the largest to the smallest were found in the ventral and dorsal attention network (vANT, dANT), default mode network (DMN), frontoparietal network (FPN), somatomotor network (Mot), visual network (Vis), and limbic network (LMB).

Figure 2A shows the whole-brain Asso distribution under a threshold of *r* = 0.2. The high Asso values were mainly distributed in bilateral superior and middle frontal gyrus, posterior inferior frontal gyrus, anterior insula, lateral parietal cortex, middle temporal cortex, posterior cingulate cortex, anterior cingulate cortex, and medial prefrontal cortex. The whole-brain cortex was then divided into seven different networks to quantitatively delineate the Asso distributions in cortical networks (Figure 2B). As a result, a gradient decrease in Asso was found from vANT that showed the highest Asso values to LMB that showed the lowest Asso values. Lower than that in vANT were the Asso values that were approximately equal in the commonly recognized association networks of dATN, DMN, and FPN, followed by the Asso values in Mot and Vis networks. Compared with the Vis network, the Mot network had higher Asso values (Figure 2B).

### Threshold Effects on Association Index

**FIGURE 3:**
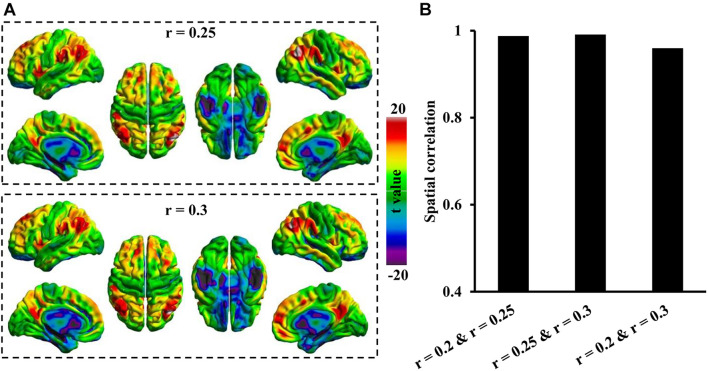
Spatial similarities of Asso distribution across different thresholds. **(A)** The whole brain Asso distribution patterns were calculated under thresholds of *r* = 0.25 and *r* = 0.3. **(B)** The spatial correlations defined using Pearson correlation coefficient across different thresholds were calculated. The Asso distribution across different threshold values showed high spatial similarities.

To further determine whether different threshold *r* values affect the Asso mapping results, the Asso maps were calculated under threshold values of 0.25 and 0.3. The results showed that Asso distribution patterns under these two threshold values were consistent with that at a threshold of 0.2 (Figure 3A). The spatial correlation analyses identified high spatial similarities across different threshold values (Figure 3B).

### Comparisons Between Association Index and Functional Connectivity Strength

**FIGURE 4:**
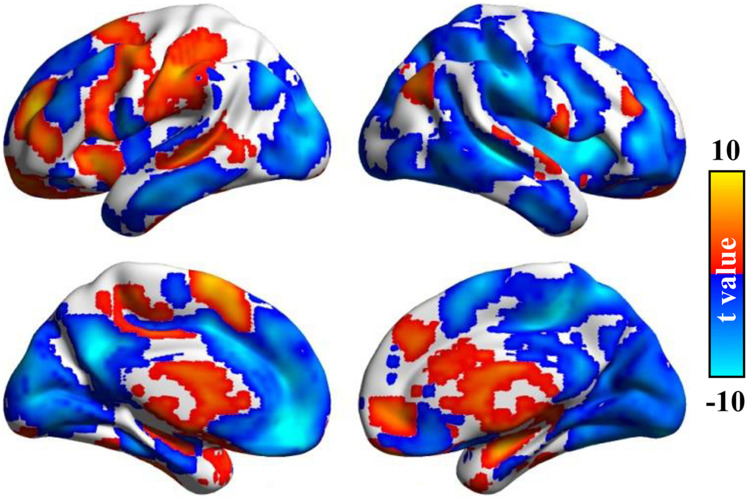
Significant differences between Asso and functional connectivity strength (FCS). The paired *t*-test was used to identify the voxel-wise differences between Asso and FCS. Significant differences between Asso and FCS maps were found.

After statistical analysis, significant differences between the Asso method we developed and the FCS method were found in different brain areas. Compared with FCS, the higher Asso was found in the bilateral thalamus and basal ganglia, bilateral parahippocampus, amygdala, left postcentral gyrus, supramarginal gyrus, anterior precuneus, posterior middle temporal sulcus, posterior superior/middle/inferior frontal gyrus, anterior middle/inferior frontal gyrus, anterior insula, supplementary motor area, right angular gyrus, superior temporal gyrus, ventral premotor cortex, anterior middle frontal gyrus, dorsomedial prefrontal cortex, anterior cingulate cortex, and medial prefrontal cortex (Figure 4). However, the lower Asso values were found in the default mode network, bilateral occipital cortex, primary visual cortex, middle/inferior temporal gyrus, posterior ventral anterior cingulate cortex, hippocampus, left superior temporal gyrus, middle insula, medial prefrontal cortex, anterior superior frontal gyrus, central middle frontal gyrus, right superior parietal lobule, supramarginal gyrus, insula, precentral/postcentral gyrus, superior frontal gyrus, anterior middle frontal gyrus, supplementary motor area, and posterior precuneus (Figure 4). These findings indicated that Asso and FCS showed different patterns to characterize brain functional architecture.

### Aging Effects Revealed by Association Index

**FIGURE 5:**
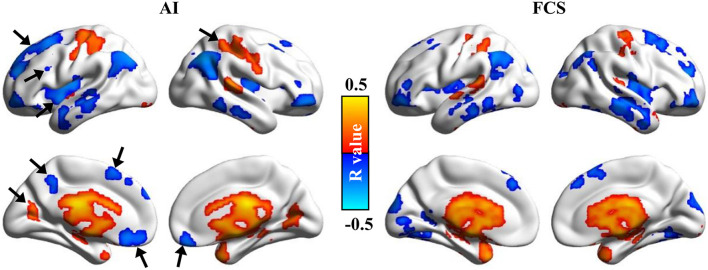
Aging effects were revealed using the Asso and FCS methods. The voxel-wise correlation analyses between ages and Asso, FCS were performed. Significantly positive and negative correlations between ages and Asso, FCS were found. We found that Asso could be more effective in identifying aging-related brain areas.

The voxel-wise correlation analyses were performed between Asso maps and age to explore whether Asso is sensitive enough to reveal the aging effects. The significantly positive correlations between age and Asso values were found in the bilateral precentral/postcentral gyrus, cuneus, parahippocampus, thalamus, basal ganglia, amygdala, and superior temporal gyrus (Figure 5). The significantly negative correlations between age and Asso values were found in the bilateral superior/middle frontal gyrus, anterior inferior frontal gyrus, middle/inferior temporal gyrus, angular gyrus, insula, subgenual anterior cingulate cortex, and left precuneus during aging (Figure 5). For comparisons, the aging effect was also analyzed using the FCS method. Although both methods identified almost identical brain areas, the Asso method discovered some new brain areas related to age effects, whereas the FCS method did not. These new brain areas included the left superior frontal gyrus, middle frontal gyrus, insula, supplementary motor area, precuneus, right superior parietal lobule, and bilateral subgenual anterior cingulate cortex (Figure 5). The results indicated that the Asso method could provide more information than the FCS method to reveal aging effects.

## Discussion

This study introduced a voxel-wise approach to map the association ability of the brain, i.e., association ability (Asso), using resting-state fMRI. The proposed method was validated by analyzing the distribution pattern in cortical networks, different threshold values, comparison with FCS, and application to identify age effects. Our findings demonstrated that Asso is a reliable method for mapping the whole brain association ability and facilitating future cognitive and clinical studies.

The spatial distribution patterns of Asso showed that the high Asso is predominantly distributed in the ventral and dorsal attention network (v/dANT), default mode network (DMN), and frontoparietal network (FPN), whereas the low Asso is mainly distributed in visual (Vis) and limbic (LMB) networks. The median *v* value is in the somatomotor (Mot) network. These findings indicated that Asso could effectively differentiate association cortex and high-order cognition-related brain areas from primary sensory- and motor-related areas. The areas belonging to v/dANT, DMN, and FPN are commonly recruited as the functional hubs of the brain and play pivotal roles in information transition and integration. For example, the vANT is specialized for the detection of behavior-related stimuli and the direction of attention to salient events, while the dANT is involved in preparing and applying goal-directed (top–down) selection for stimuli and responses (Corbetta and Shulman, 2002; Wang et al., 2016a; Wu Y. et al., 2016). DMN, which is composed of several frontal, parietal, and temporal areas, shows increased activity under rest but decreased activity under task and is reported to be mainly involved in self-reference and social cognition processing (Raichle et al., 2001; Wang J. et al., 2019; Wang L. et al., 2019). FPN, a central executive network, is responsible for high-level cognitive functions and external information processing (Menon, 2011; Wang et al., 2018). The v/dANT, DMN, and FPN constitute the unifying triple network modes essential for high-order cognitive functions (Menon, 2011). The vANT corresponding to the salience network keeps homeostatic interoception and external stimulus to accommodate the dynamic interaction between the internal self-perception and external orient stimulus. Thus, vANT is considered to be a hub connecting DMN and FPN or dANT for information switch. The finding in our study that vANT has the highest Asso values corroborates the prior knowledge on the critical role of vANT.

Both FCS and FCD are widely used approaches to define the brain hubs to characterize the brain association ability. The FCS is defined as degree centrality by counting the number of voxels above a predefined threshold but not considering the interconnections of the connected voxels of the specific voxel (Buckner et al., 2009). For the Asso method, it not only calculates the number of functional connections above a predefined threshold for a specific voxel but also computes the number of interconnections of the connected neighbors of the specific voxel. The difference of the numbers of connections was defined as Asso. Thus, Asso characterizes the necessity of a voxel to form a connection for two functionally unconnected voxels. The voxel-level comparisons revealed significant differences between Asso and FCS in our study, indicating that Asso and FCS have their own advantage in characterizing the association for different brain networks. Differently, FCD is measured by counting the number of neighboring voxels of which functional connectivity strength is higher than a predefined threshold using the regional growth method (Tomasi and Volkow, 2010). The hubs with high FCD values identified by FCD mapping are primarily distributed in the posterior cingulate/ventral precuneus, middle cingulate cortex, cuneus, calcarine cortex, and inferior parietal regions. However, FCD mapping did not report the commonly recognized hub areas, especially in frontal areas. Given the tremendous methodological differences of FCD compared with FCS and Asso, FCD may be more suitable to define the hubs that show both high local and global connections.

The aging-related brain areas identified with the Asso method in our study include all the relevant regions reported by previous studies, indicating that Asso may gain the advantages of FCS and FCD minimally for aging study. Normal aging is associated with cognitive decline affecting attention, memory, and executive functions (Hedden and Gabrieli, 2004; Whalley et al., 2004). The aging effects on resting-state functional connectivities within DMN and dANT have been well documented (Ferreira and Busatto, 2013). A recent study using the FCS method found decreased FCS in dANT and DMN and increased FCS in the somatomotor network, anterior insula, and lateral prefrontal cortex (Li et al., 2020). Nevertheless, the enrolled number of subjects is small. Likewise, FCD mapping revealed that aging was also associated with decreased FCD in DMN and dANT and with increased FCD in somatosensory and subcortical networks (Tomasi and Volkow, 2012). The findings in the two aforementioned studies about aging are consistent with our results identified using the Asso method. Thus, the Asso method alone exhibits an integrative potential in pinpointing brain regions related to the association ability. Moreover, the aging effects that we investigated using the FCS method in our study failed to locate DMN that has been widely reported during aging, suggesting that Asso may be a more reliable approach to delineate the changed association ability of the brain.

There are some limitations to our study. First, we only studied aging effects using the Asso method. Whether or not Asso can also identify development patterns need further validation. Second, the Asso validity could be verified reversely from the aspect of disconnections in the brain diseases that show symptoms of disrupted functional connectivities. Third, a threshold was applied during Asso calculation to exclude weak connections caused by noise, even though the threshold selection is a common issue for all the FCS, FCD, and our methods.

## Data Availability Statement

The raw data supporting the conclusions of this article will be made available by the authors, without undue reservation.

## Ethics Statement

The studies involving human participants were reviewed and approved by all subjects were given informed written consent approved by the Institutional Review Board. The patients/participants provided their written informed consent to participate in this study.

## Author Contributions

All authors listed have made a substantial, direct and intellectual contribution to the work, and approved it for publication.

## Conflict of Interest

The authors declare that the research was conducted in the absence of any commercial or financial relationships that could be construed as a potential conflict of interest.

## Publisher’s Note

All claims expressed in this article are solely those of the authors and do not necessarily represent those of their affiliated organizations, or those of the publisher, the editors and the reviewers. Any product that may be evaluated in this article, or claim that may be made by its manufacturer, is not guaranteed or endorsed by the publisher.

## Footnotes

1
http://fcon_1000.projects.nitrc.org/indi/retro/sald.html
